# The Power of Posing: Do Body Display Instructions Have an Impact on Behavior in Daily Life?

**DOI:** 10.1002/brb3.70643

**Published:** 2025-06-18

**Authors:** Mai Bjørnskov Mikkelsen, Emma Elkjær, Douglas S. Mennin, Johannes Michalak, Mia S. O'Toole

**Affiliations:** ^1^ Department of Psychology and Behavioural Sciences Aarhus University Aarhus Denmark; ^2^ Department of Counseling and Clinical Psychology Teachers College, Columbia University New York New York USA; ^3^ Department of Psychology and Psychotherapy Witten/Herdecke University Witten Germany

**Keywords:** action tendencies, body displays, emotions, power posing

## Abstract

**Introduction:**

The present study investigated the effects of expansive and contractive body display instructions on adaptive behavior and affect within the context of personally relevant behavioral goals set in daily life. The moderating effects of motivational traits and symptoms of psychopathology were explored.

**Methods:**

A sample of 127 adults identified personally relevant and challenging actions they wanted to take during each week for 12 consecutive weeks. Before taking action, participants were randomly assigned to listen to instructions for one of four body manipulations: (1) expansive, (2) contractive, (3) neutral, or (4) active control (i.e., walking in place). The behavioral outcome was the extent to which participants took the wanted action, and the affective outcomes were emotions, action tendencies, and appraisals.

**Results:**

The results showed no effect of body displays on behavior (*d *= 0.06) nor affect (*d*s = 0.02–0.06). Neither motivational traits nor symptoms of psychopathology moderated the effects of body displays on behavior and affect. Changes in action tendencies (i.e., avoid reward, approach reward) and appraisals (i.e., appraised difficulty, importance, and self‐efficacy) predicted taking action as planned.

**Conclusion:**

The results indicate that the body display instructions under investigation did not have an effect on taking action or associated affect. Such findings are more consistent with theories suggesting variability in the association between the motor system, emotions, and behavior across contexts than theories suggesting invariant associations. Future research may investigate individualized body displays and whether the effects of body manipulations vary systematically with features of the context.

## Graphical abstract

This study is among the first to test how body manipulation instructions influence everyday actions. The findings indicate no consistent effects of body manipulation instructions on behavior or emotion, suggesting that the effects of body displays on behavior and affect may be variable.

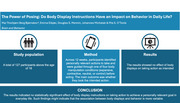

## 1 Introduction

A growing body of theoretical and empirical work argues for the embodied nature of cognition, emotion, and behavior (Michalak et al. 2015; Niedenthal et al. 2005; Winkielman et al. 2015). Theories of embodiment and embodied cognition encompass a range of diverse theoretical perspectives converging on the basic idea that bodily processes and movement have an impact on how we think, feel, and act (Damasio 1999; Gallagher 2023; Shapiro 2011; Wilson and Golonka 2013; Wilson 2002; Winkielman et al. 2015). One way in which the body may shape cognition, emotion, and behavior is through posture and movement (Cuddy et al. 2018, 2015; Matheson and Barsalou 2018; Michalak et al. 2015; Niedenthal et al. 2005; Welker et al. 2013). For example, Körner and Schütz (2020) and Körner et al. (2022) proposed the dominance–prestige framework to explain how certain body displays become associated with specific concepts related to dominance and prestige through evolution and associative learning. Specifically, the dominance–prestige framework proposes that *expansive body displays* (i.e., open, upright, and powerful postures, making the body appear taller and wider) are associated with positive and adaptive behavioral outcomes (e.g., competence and accomplishment), while *contractive body displays* are associated with negative and maladaptive behavioral outcomes (e.g., failure and ineptitude). According to the dominance–prestige framework, activation of one aspect of a concept, such as the mental, behavioral, or bodily state, will lead to the activation of the other aspects (Körner et al. 2022; Körner and Schütz 2020). As such, assuming an expansive or contractive body display will activate associated concepts, leading to mental and behavioral inclinations consistent with the activated concept (e.g., feeling inferior when assuming a contractive display; Körner et al. 2022).

Findings from studies of the impact of the motor system on various emotional (e.g., felt power) and behavioral (e.g., risk‐taking) outcomes have been mixed (Carney et al. 2015; Cesario and McDonald 2013; Cuddy et al. 2018; Elkjær, Mikkelsen, Tramm, et al. 2022; Garrison et al. 2016; Gronau et al. 2017; Jonas et al. 2017; Metzler and Grèzes 2019; Metzler et al. 2023; Ranehill et al. 2015; Simmons and Simonsohn 2017; Smith and Apicella 2017). Two recent meta‐analyses have synthesized the existing evidence on differences between expansive and contractive displays and found significant differences across studies on self‐reported and behavioral outcomes but no significant differences across studies on hormones and physiological outcomes (Elkjær et al. 2022a; Körner et al. 2022). However, Körner et al. (2022) concluded that the effects on behavioral outcomes were not robust. In addition, both meta‐analyses drew attention to the small number of studies that included control groups without manipulations of body displays or with control manipulations (e.g., neutral body displays; Elkjær, Mikkelsen, Tramm, et al. 2022; Körner et al. 2022). Given the uncertain effects on behavioral outcomes and the lack of control groups, more research is clearly needed to clarify the effects of manipulations of the motor system on affect and behavior.

In addition to the uncertainty around the effects on behavioral outcomes and the scarcity of studies with control groups, two prominent limitations of previous research include the lack of personal relevance of experimental settings and the lack of consideration of individual differences (Elkjær et al. 2022a; Mikkelsen et al. 2024). Starting with personal relevance, most studies have investigated body displays in experimental settings where participants have been exposed to predetermined tasks with little personal relevance (e.g., a mock job interview; Cuddy et al. 2015; Elkjær et al. 2022a; Huang et al. 2011). Whether findings from such studies generalize to personally relevant behavioral goals in everyday life remains to be determined. Turning to individual differences, both motivational dispositions and symptoms of psychopathology may be hypothesized to play a role in the effect of body manipulations on behavior (Mikkelsen et al. 2024). Concerning motivational dispositions, research suggests that there may be individual differences in the extent to which people are motivated to engage in approach behavior and avoidance behavior (Carver and White 1994; Locke and Braver 2008; Monni et al. 2020). Such differences may explain why some people are more inclined to take action to achieve a personally relevant goal. However, research on body manipulation has rarely taken these dispositions into account. Motivation is also a factor in psychopathology, where distinct patterns of motivation have been observed in conditions like depression and anxiety (Michalak et al. 2006; Trew 2011; Winch et al. 2015). In addition, research indicates alterations in the motor system in individuals with depression and anxiety (e.g., Elkjær et al. 2022b; Mikkelsen and O'Toole 2023). However, findings from studies of the impact of body manipulations on individuals with psychopathology have been mixed (Davis et al. 2017; Flack et al. 1999; Michalak et al. 2018; Michalak et al. 2014; Wilkes et al. 2017). Therefore, it remains to be determined whether and how motivational dispositions and symptoms of psychopathology affect the impact of body manipulations on affect and behavior.

### 1.1 The Present Study

In the present study, we extend previous research by examining the impact of expansive and contractive body display instructions on both adaptive behavior (i.e., taking action to reach a personally relevant goal) and affect (i.e., appraisals, emotions, and action tendencies) within the context of personally relevant behavioral goals set in daily life. The study is an Ecological Momentary Intervention (EMI) study, which refers to interventions “that are provided to people during their everyday lives (i.e., in real time) and in natural settings (i.e., real world)” (Heron and Smyth 2010, 1). Based on previous literature, the following preregistered hypotheses were developed and tested in the present study:

H_1_: Participants who adopt an expansive posture will be more likely to take action, report higher levels of momentary approach motivation and lower levels of avoidance/withdrawal motivation, report higher levels of momentary perceived self‐efficacy, and report lower levels of momentary perceived difficulty of taking action compared to participants who adopt a contractive posture and to participants in active (i.e., running in place) and neutral control conditions (i.e., adopting a neutral posture).

H_2_: Participants who adopt a contractive posture will be less likely to take action, report lower levels of momentary approach motivation and higher levels of avoidance/withdrawal motivation, report lower levels of momentary perceived self‐efficacy, and report higher levels of momentary perceived difficulty in taking action compared to participants who adopt a contractive posture and to participants in active and neutral control conditions.

H_3_: The effect of expansive and contractive postures on behavior (i.e., taking action) will happen as a result of changes in momentary motivational state, momentary emotional state, momentary perceived self‐efficacy, and momentary perceived difficulty of taking action.

## 2 Materials and Methods

The study was preregistered at aspredicted.org (#51661) and ethically approved by the local Institutional Review Board at Aarhus University (#2020‐88). The following amendments were made to the original preregistration: (1) the aim was to include 180 participants, but due to a high number of dropouts only 127 participants were included; (2) participants were excluded if they could not complete the body manipulations; and (3) exploratory analyses were conducted to assess whether motivational dispositions and symptoms of psychopathology moderated the effects of body manipulation instructions on behavior and affect.

### 2.1 Participants

Participants were recruited through fliers and advertisements online. To be included, participants had to be above the age of 18 years and proficient in the Danish language. Participants were excluded if they were unable to complete the body manipulations, e.g., due to pain. Participants were compensated with 200 DKK (∼$29) for completing the study.

### 2.2 Procedure

**FIGURE 1:**
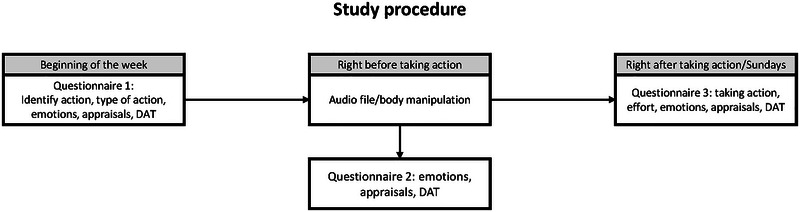
Overview of the weekly study procedure. For each week, participants were randomly assigned to one of four body manipulations: (1) expansive posture, (2) contractive posture, (3) neutral posture, and (4) active control group. DAT, Depicted Action Tendency questionnaire (O'Toole and Mikkelsen 2021).

Participants received oral and written information about the project and provided online informed consent. After completing baseline questionnaires, they were enrolled in the EMI part of the study. For the EMI, participants received links to three questionnaires via SMS every Monday for 12 consecutive weeks. The first questionnaire prompted participants to identify a personally relevant and valued action of moderate difficulty (rated ≥ 4 on a 7‐point Likert scale ranging from 1 = *Not at all* to 7 = *Very much*) that they wanted to take during the week (T_1_). Participants were asked to choose an action that was difficult to take because either participants were (1) nervous or afraid of taking action (*avoidance conflict*; e.g., going on a first date, going for a job interview) or (2) not in the mood or could not muster the energy to take action (*withdrawal conflict*; e.g., working out, completing a work assignment). The second questionnaire was to be filled in as close in time to when the participants planned to take action as possible (T_2_) and contained a link to a 3‐min audio file with instructions for an expansive posture (EXP), a contractive posture (CON), to walk in place (active control [ACO]), or a neutral posture (NEU). The participants were randomly assigned to receive one of the four instructions each week with the restriction that all participants received each instruction three times during the 12‐week EMI period. Scripts for the instructions can be found in the Supporting Information. The third questionnaire was filled out right after taking action or on Sundays if the participant did not take action (T_3_). As a manipulation check, participants were asked to indicate whether they accessed and listened to the audio file or not (T_3_) and the extent to which they made an effort to take action on a 5‐point Likert scale (1 = *No effort*; 5 = *Maximum effort*). An overview of the study procedure is presented in Figure 1.

### 2.3 Measures

#### 2.3.1 Baseline Measures

*Depression, anxiety, and stress* for the previous 2 weeks were measured using the short‐form version of the Depression Anxiety Stress Scale (DASS‐21; Henry and Crawford 2005; Lovibond et al. 1995; depression: *α* = 0.88; anxiety: *α* = 0.81; stress: *α* = 0.82). High scores on the subscales indicate more symptoms of depression, anxiety, and stress, respectively.

*Environmental reward sensitivity* for the previous 2 weeks was assessed using the Environmental Reward Observation Scale (EROS; Armento and Hopko 2007; *α* = 0.82). High scores on the EROS indicate greater environmental reward sensitivity.

*Trait‐level behavioral inhibition and activation* were measured using the BIS/BAS scale (Carver and White 1994). The BIS/BAS yields scores for four subscales: the behavioral inhibition subscale (*α* = 0.76) and three behavioral activation subscales: the Drive subscale (*α* = 0.79), the Fun Seeking subscale (*α* = 0.60), and the Reward Responsiveness subscale (*α* = 0.59). High scores on the BIS and BAS subscales indicate greater behavioral inhibition and greater behavioral activation, respectively.

#### 2.3.2 Outcomes

*Taking action* was assessed at T_3_. Participants rated the extent to which they took action as planned on a 5‐point Likert scale (1 = *Not at all*; 5 = *Completely as planned*).

*Momentary action tendencies* were assessed by participants’ ratings on the Depicted Action Tendency instrument (DAT) (O'Toole and Mikkelsen 2021) at T_1_, T_2_, and T_3_ on 5‐point Likert scales (1 = *Not at all*; 5 = *Very much*; see Supporting Information). Mean scores for avoid threat, avoid reward, approach threat, and approach reward action tendencies at each time point were calculated.

*Momentary emotions* were assessed by participants’ ratings of seven positive (happiness, enthusiasm, contentment, amusement, curiosity, pride, and gratitude) and eight negative (sadness, disgust, nervousness, fear, frustration, guilt, anger, and shame) emotions at T_1_, T_2_, and T_3_ on 5‐point Likert scales (1 = *Not at all how I feel;* 5 = *Very much how I feel*). Based on emotion ratings, scores of total positive and total negative emotions were computed for each time point.

*Appraisals* of the importance and difficulty of completing the task, of self‐efficacy, and of change in mood were assessed at T_1_ and T_2_. Participants rated the extent to which they found the action difficult and important, and they rated their self‐efficacy on a 7‐point Likert scale (1 = *Not at all*; 7 = *Very much*). Participants rated expected mood change upon completing the action on a 7‐point Likert scale (−3 = *Negative impact*; 3 = *Positive impact*).

### 2.4 Statistical Analyses

Following the preregistered analytic plan, multilevel analyses (MLMs) were conducted to test the three hypotheses. Only complete weeks, that is, weeks where participants reported actually listening to the sound files, were included in the MLMs. All MLMs were computed with random intercepts and with time points (Level 1) nested within individuals (Level 2). The MLMs are specified in the Supporting Information.

Syntaxes are available at the Open Science Framework using the link: https://osf.io/8wn5g/?view_only=ff06b14e68ac42688dbb4137ab874a27.

## 3 Results

### 3.1 Participant Characteristics and Planned Actions

A sample of 270 participants was recruited. A total of 143 participants were excluded because they completed less than 3 weeks of the EMI part of the study. The final sample included in the study consisted of 127 participants. A post hoc sensitivity analysis indicated that the study was powered to detect effects of Cohen's *d* = 0.30 (*p* = 0.05, *α* = 0.80; see Supporting Information). The sample was predominantly female (84.3% female, 15% male, 0.8% nonbinary), and the mean age of participants was 30.1 (SD = 10.1, range: 19–63).

On average, participants completed 11 (93.3%) of the weeks (i.e., completing at least the first questionnaire and last questionnaires). For the majority of the completed weeks, participants listened to the body manipulation instructions (67.7%) and reported completing the planned action (82.7%). The mean rated difficulty of planned actions was 5.6 (SD = 1.0). Most actions could be categorized as withdrawal conflicts (59.1%), while the remaining were fear avoidance conflicts (28.2%), mixed conflicts (12.2%), or uncategorized (0.5%). The median rating of effort was 4, indicating that participants generally made an effort to complete the planned action.

### 3.2 Confirmatory Analyses

#### 3.2.1 Do Body Manipulations Affect Planned Actions and Affective Outcomes?

Results revealed no significant group differences between body display instructions for taking action^1^ nor for any affective outcome. Hypotheses 1, 2, and 3 were thus not confirmed (See Table 1 and Table 2)^2^.

**TABLE 1:** Outcome descriptives.

	Planning action (T_1_)	Pre‐action (T_2_)				Post‐action (T_3_)			
		EXP	CON	ACO	NEU	EXP	CON	ACO	NEU
	*M* (SD)	*M* (SD)	*M* (SD)	*M* (SD)	*M* (SD)	*M* (SD)	*M* (SD)	*M* (SD)	*M* (SD)
Taking action	—	—	—	—	—	4.0 (1.2)	3.9 (1.2)	4.0 (1.3)	3.9 (1.3)
Positive emotions	15.3 (6.4)	15.5 (6.7)	15.6 (6.9)	15.2 (6.3)	15.4 (6.3)	14.4 (4.8)	14.3 (4.4)	14.4 (4.2)	14.2 (4.7)
Negative emotions	15.5 (6.1)	13.7 (5.4)	14.8 (6.3)	14.3 (5.9)	14.1 (5.9)	14.4 (3.8)	14.6 (3.8)	14.4 (3.7)	14.4 (4.0)
DAT—avoid threat	2.5 (1.3)	2.2 (1.1)	2.3 (1.3)	2.4 (1.3)	2.2 (1.2)	1.7 (0.9)	1.8 (1.1)	1.7 (1.0)	1.6 (0.9)
DAT—approach threat	2.0 (0.9)	2.0 (0.8)	2.0 (0.8)	2.0 (0.9)	1.9 (0.8)	2.0 (0.9)	2.0 (0.9)	2.0 (0.9)	2.0 (0.8)
DAT—avoid reward	2.4 (1.2)	2.1 (1.2)	2.2 (1.2)	2.1 (1.2)	2.1 (1.2)	1.7 (1.0)	1.8 (1.1)	1.8 (1.1)	1.8 (1.1)
DAT—approach reward	1.9 (1.1)	2.1 (1.1)	2.1 (1.1)	2.1 (1.1)	2.0 (1.1)	2.6 (1.3)	2.5 (1.3)	2.5 (1.3)	2.6 (1.3)
Appraisals Difficulty Importance Self‐efficacy Expected mood change	5.6 (1.0) 5.9 (1.2) 4.6 (1.4) 5.5 (1.5)	4.8 (1.5) 5.8 (1.2) 5.1 (1.6) 5.5 (1.4)	5.1 (1.3) 5.8 (1.3) 5.1 (1.5) 5.4 (1.5)	4.8 (1.4) 5.7 (1.2) 5.1 (1.6) 5.6 (1.4)	4.9 (1.3) 5.8 (1.3) 5.2 (1.4) 5.6 (1.4)	— — — —	— — — —	— — — —	— — — —

*Note*: Means were calculated by averaging scores across all experience‐sampling measurement points.

Abbreviations: ACO, active control; CON, contractive posture; EXP, expansive posture; NEU, neutral control.

**TABLE 2:** Results from multilevel models assessing the impact of time and body display instructions on planned action and affective outcomes.

	Time differences (T_1_ to T_2_)			Group differences at T_3_			Group × time differences (T_1_–T_2_)			Group × time differences (T_1_–T_3_)		
	*F*	*p*	*d*	*F*	*p*	*d*	*F*	*p*	*d*	*F*	*p*	*d*
Taking action	—	—	—	0.81	0.490	0.06	—	—	—	—	—	—
Positive emotions	0.28	0.599	0.02	0.47	0.703	0.05	0.85	0.466	0.04	1.86	0.134	0.06
Negative emotions	**44.25**	**< 0.001**	**0.31**	0.17	0.918	0.03	0.78	0.506	0.04	0.21	0.888	0.02
DAT—avoid threat	**29.94**	**< 0.001**	**0.26**	0.49	0.691	0.05	0.52	0.666	0.03	0.13	0.941	0.02
DAT—Approach threat	0.12	0.724	0.02	0.31	0.819	0.04	0.73	0.533	0.04	0.27	0.846	0.02
DAT—avoid reward	**36.03**	**< 0.001**	**0.28**	0.07	0.977	0.02	0.88	0.449	0.04	0.38	0.768	0.03
DAT—approach reward	**17.97**	**< 0.001**	**0.20**	0.27	0.849	0.04	1.64	0.177	0.06	0.54	0.655	0.03
Appraisals Difficulty Importance Self‐efficacy Expected mood change	**200.00** **14.58** **105.37** 0.01	**< 0.001** **< 0.001** **< 0.001** 0.929	**0.67** **0.18** **0.48** < 0.01	— — — —	— — — —	— — — —	2.20 0.46 0.78 0.18	0.086 0.711 0.505 0.908	0.07 0.03 0.04 0.02	— — — —	— — — —	— — — —

*Note*: Statistically significant results at *p* < 0.05 are indicated in bold.

Abbreviations: DAT, Depicted Action Tendencies; T_1_, planning action; T_2_, right before taking action; T_3_, right after taking action or at the end of the week.

Post hoc paired comparisons of groups were conducted using MLMs, which included only two groups at a time. The results suggested no significant group differences for any outcome at T_3_ (ESs ranging from *d *≤ 0.01–0.14) and for changes in any outcome from T_1_ to T_3_ (ESs ranging from *d *≤ 0.01–0.13). For changes in outcomes from baseline (T_1_) to before taking action (T_2_), participants in the expansive condition experienced a greater decrease in appraised difficulty (mean change = −0.84) compared to participants in the contractive condition (mean change = −0.5; *p* = 0.009, *d* = 0.18), and participants in the expansive condition experienced a greater increase in approach reward action tendencies (mean change = 0.3) than participants in the neutral condition (mean change = 0.07; *p* = 0.030, *d* = 0.15). No other significant group differences were identified for changes in outcomes from T_1_ to T_2_ (ESs ranging from *d *≤ 0.01–0.11).

### 3.3 Exploratory Analyses

#### 3.3.1 Do Changes in Affective Outcomes Affect Planned Actions?

As the body display instructions had no statistically significant effect on taking action, multilevel models were conducted to investigate whether changes in affective outcomes had an effect on taking action, disregarding body manipulations. The results indicated significant reductions from T_1_ to T_2_ in negative emotions, avoid‐threat action tendencies, avoid‐reward action tendencies, difficulty, and importance of the action, as well as significant increases in approach‐reward action tendencies and self‐efficacy. Analyses further showed that reductions in avoid reward action tendencies and perceived difficulty of taking action were associated with higher ratings of taking action according to one's plan (see Table 3). Furthermore, increases in approach reward action tendencies, importance, and self‐efficacy were associated with higher ratings of taking action according to one's plan^3^.

**TABLE 3:** Results from multilevel models assessing changes in affective outcomes as predictors of taking action.

	Change in affective outcomes (T_1_–T_2_) predicting taking action (T_3_)			
	*F*	*t*	*p*	*d*
Negative emotions	3.57	−1.89	0.059	0.13
DAT—Avoid threat	1.71	−1.31	0.192	0.09
DAT—Avoid reward	**18.98**	**−4.36**	**< 0.001**	**0.30**
DAT—Approach reward	**25.05**	**5.00**	**< 0.001**	**0.35**
Appraisals Difficulty Importance Self‐efficacy	**39.87** **24.80** **60.82**	**−6.31** **4.98** **7.80**	**< 0.001** **< 0.001** **< 0.001**	**0.44** **0.34** **0.54**

*Note*: Statistically significant results at *p* < 0.05 are indicated in bold.

Abbreviations: DAT, Depicted Action Tendencies; T_1_, planning action; T_2_, right before taking action; T_3_, right after taking action or at the end of the week.

#### 3.3.2 Moderator Analyses

Results from analyses conducted to explore the proposed moderators (i.e., BIS/BAS, EROS, and DASS) are provided in Table 4. The results revealed no significant effects of the proposed moderators.

**TABLE 4:** Results from analyses investigating the moderating effect of motivational traits and symptoms of psychopathology on the effect of body display instructions on taking action.

	Manipulation × moderator predicting taking action at T_3_		
	*F*	*p*	*d*
BIS/BAS—inhibition	1.07	0.361	0.07
BIS/BAS—drive	0.80	0.492	0.06
BIS/BAS—fun seeking	1.97	0.117	0.10
BIS/BAS—reward responsiveness	0.33	0.805	0.04
EROS	2.40	0.066	0.11
DASS—stress	1.23	0.299	0.08
DASS—anxiety	1.88	0.131	0.10
DASS—depression	2.74	0.042	0.11

*Note*: Statistically significant results at an adjusted *p‐*level of < 0.017 (0.05/3) are indicated in bold.

Abbreviations: BAS, Behavioral Activation Scale; BIS, Behavioral Inhibition Scale; DASS, Depression Anxiety Stress Scale; EROS, Environmental Reward Observation Scale; T_3_, right after taking action or at the end of the week.

## 4 Discussion

The present study found no overall significant effects of body manipulation instructions on taking action to achieve a personally relevant behavioral goal or associated affect. As such, our hypotheses that (i) participants who are instructed to adopt an expansive posture would be more likely to take action and report more favorable affective outcomes than participants who are instructed to adopt a contractive posture and participants in control conditions, and (ii) participants who are instructed to adopt a contractive posture would be less likely to take action and report less favorable affective outcomes than participants who are instructed to adopt an expansive posture and participants in the control condition, were not confirmed. Pairwise comparisons revealed a few small but significant differences in change in affective outcomes from baseline to before taking action. Given that the overall tests of group differences were nonsignificant, these pairwise comparisons should be interpreted with caution. Finally, the present findings suggested that neither motivational dispositions nor symptoms of psychopathology significantly moderated the effects of body manipulations on taking action.

The findings may be taken to be inconsistent with theories such as the dominance–prestige framework proposing relatively stable associations between body displays, affect, and behavior (Körner et al. 2022). Instead, the findings may (1) indicate that body displays have a smaller or no effect on affect and behavior, (2) be a product of the present study's methods, or (3) suggest that the association between body displays, affect, and behavior is variable.

Starting with the first interpretation, it may be the case that body displays have a smaller or no effect on affect and behavior. However, previous studies have identified effects ranging from negligible to large (Hedges’ *g *≤ 0.001; 1.13, Elkjær, Mikkelsen, Tramm, et al. 2022; *g* = 0.01; 3.9; Körner et al. 2022), suggesting that it may be too conservative to claim that body displays *generally* have a smaller or no effect on affect and behavior. The heterogeneity in effects obtained in previous studies indicates the presence of moderating factors such as study design and context.

Turning to the second interpretation, the findings may be a product of the present study's methods. The small sample size and the nature and duration of the body displays may have limited the power to detect differences between groups. In terms of the manipulation instructions employed, they are consistent with previous studies on body manipulations (Elkjær et al. 2022a; Körner et al. 2022), investigating single, brief, and overstated body displays. However, such body displays may bear little resemblance to the everyday body displays that people typically assume, which may be less exaggerated and have a duration that is adapted to the context. It may be the case that exaggerated body displays and displays of short duration, such as those employed in previous laboratory studies and in the present study, have a smaller effect when investigated in the context of daily life than in experimental contexts. In addition, although we used manipulation checks to confirm that the participants listened to the body display instructions, we cannot determine whether the participants followed the instructions. Future research may benefit from investigating body displays that more closely resemble participants’ natural body displays and from ensuring that participants follow body display instructions.

Concerning the last interpretation, our findings may be taken as an indication of the association between body displays, affect, and behavior being variable. This would be consistent with contemporary emotion theories, suggesting that there may be variability in the association between body displays, affect, and behavior across different contexts and based on individual goals within that context (Barrett 2017a; Lindquist 2013; Mikkelsen and O'Toole 2023; Moors et al. 2013). For example, several contemporary emotion theories (e.g., Barrett 2017a, 2017b; Moors et al. 2013) suggest that many different bodily displays and behavioral inclinations may be associated with the same emotional state (e.g., straightening *and* slumping one's shoulders when fearful) and that one type of bodily display may be associated with several different emotional states and behavioral inclinations (e.g., tense and rigid posture when angry *and* when excited). This explanation aligns well with the substantial variability in the effects of body displays across studies found in the meta‐analyses by Elkjær, Mikkelsen, Tramm, et al. (2022) and Körner et al. (2022). Investigating whether the effects of body displays on emotional and behavioral outcomes vary systematically with features of the context and individual goals, needs, and wants represents a promising avenue for future research.

Turning to the investigated moderators, the findings suggest that neither motivational dispositions nor symptoms of psychopathology moderated the effects of body displays on taking action in everyday life. These null findings for motivational dispositions align with our recent experimental study (Mikkelsen et al. 2024). However, concerning symptoms of psychopathology, the experimental study found a moderating effect of symptoms of depression for several affective outcomes (Mikkelsen et al. 2024), while the present EMI study did not. As neither the experimental study nor the EMI study was conducted with participants diagnosed with depression, and findings from studies exploring body manipulations in the context of depression have been mixed (Davis et al. 2017; Elkjær et al. 2022b; Flack et al. 1999; Michalak et al. 2018; Michalak et al. 2014), more research is clearly needed to understand the effects of body displays in individuals with depression.

Lastly, disregarding body manipulations, the present findings suggest that changes in appraisals and action tendencies were associated with taking action. Such findings may be interpreted within the context of contemporary appraisal theories, which suggest that appraisals are the primary causal mechanism that produce changes in emotion components, including action tendencies, behavior, and feelings (Moors et al. 2013). However, it should be noted that the present study was not designed to evaluate the causal relationship between the affective and behavioral outcomes.

### 4.1 Limitations

First, we did not meet the preregistered sample size. However, a post hoc sensitivity analysis indicated that the study was powered to detect small‐to‐medium effects (*d *= 0.30).

Second, external circumstances beyond the participants’ control may have prohibited them from completing the planned actions. For example, a participant may have planned to have a difficult conversation with a work colleague and then discovered that their colleague was out sick from work. In such cases, participants were asked to report that they did not complete the action.

Third, the present study assessed the effects of body manipulation *instructions* on taking action and associated affective outcomes. Given the method applied (i.e., experience sampling in the everyday life of participants), it was not possible to verify that participants followed the body display instructions.

### 4.2 Conclusion

The results of the present study indicated no statistically significant effect of body display instructions on taking action to achieve a personally relevant goal or on associated affect. Such findings might indicate that body displays have little or no stable effect on affect and behavior and that the association between body displays, affect, and behavior is more variable.

## Author Contributions

**Mai Bjørnskov Mikkelsen**: conceptualization, methodology, investigation, formal analysis, data curation, writing – original draft, writing – review and editing. **Emma Elkjær**: conceptualization, investigation, writing – review and editing. **Douglas S. Mennin**: conceptualization, methodology, writing – review and editing. **Johannes Michalak**: conceptualization, methodology, writing – review and editing. **Mia S. O'Toole**: conceptualization, methodology, investigation, supervision, funding acquisition, writing – review and editing.

## Ethics Statement

The study was preregistered (Aspredicted: #51661) and ethically approved by the local Ethics Committee at Aarhus University (#2020‐88). The procedures used in this study adhere to the Declaration of Helsinki.

## Consent

All participants provided informed consent before participating.

## Conflicts of Interest

The authors declare conflicts of interest.

## Peer Review

The peer review history for this article is available at https://publons.com/publon/10.1002/brb3.70643

## Supporting information




**Supporting Material**: brb370643‐sup‐0001‐SuppMat.docx

## Data Availability

The data that support the findings of the study are available on request from the corresponding author. The data can be shared upon completion of a data‐sharing agreement. Syntaxes will be made available at the Open Science Framework using the link: https://osf.io/8wn5g/?view_only=ff06b14e68ac42688dbb4137ab874a27 upon acceptance of the manuscript.

## Endnotes

1 Robustness analyses indicated that the main null findings for taking action remained robust when investigating different types of conflicts (i.e., taking action when afraid to do so and taking action when not feeling in the mood to do so) separately, when recoding taking action into a binary variable (i.e., 1 = not taking action; 2–5 = taking action), when omitting participants who indicated having knowledge of power posing, and when omitting participants who did not complete all weeks of experience sampling.

2 After the first week, participants may have become susceptible to demand characteristics. Thus, analyses were performed for the first week only. The results indicated no effect of body display instructions on taking action in a multilevel model, *F* = 0.14, *p* = 0.937, *d* = 0.08. Furthermore, the results indicate no effect of body display instructions on 1) any of the affective outcomes at T_3_, 2) changes in any of the outcomes from T_1_ to T_2_, and 3) changes in any of the outcomes from T_1_ to T_3_. The only exception was for approach reward, where there was a significant effect of body displays on change from T_1_ to T_2_, *F* = 4.40, *p* = 0.006, *d* = 0.43. Hence, results from Week 1 do not substantially differ from the general results.

3 Robustness analyses indicated that including weeks where participants did not complete the audio file did not alter the findings, with the exception that the results for change in negative emotions became statistically significant (*F* = 4.04, *t* = −2.01, *p* = 0.045, *d* = 0.13).
