# Supplementary material for: The Power of Posing: Do Body Display Instructions Have an Impact on Behavior in Daily Life?
Source: Brain Behav. 2025 Jun 18;15(6):e70643. doi: 10.1002/brb3.70643 (PMC12177200; doi:10.1002/brb3.70643)
Supplement: Supplementary file 1 — Supporting Material: brb370643‐sup‐0001‐SuppMat.docx [file BRB3-15-e70643-s001.docx]

**The Power of Posing: Do Body Display Instructions Have an Impact on Behavior in Daily Life?**

**Supplementary information on methods and results**

Mai Bjørnskov Mikkelsen^1^, Emma Elkjær^1^, Douglas S. Mennin^2^, Johannes Michalak^3^ & Mia S. O’Toole^1^

Author note

^1^Dept. of Psychology and Behavioural Sciences, Aarhus University

^2^Department of Counseling and Clinical Psychology, Teachers College, Columbia University

^3^Dept. of Psychology and Psychotherapy, Witten/Herdecke University

**Scripts for body manipulations.**

***Expansive [EXP] body manipulation:***

*Start by standing up. Be sure to stand up straight [20 s. PAUSE]. Don’t slouch. Make sure to keep your shoulders pulled back. Stand as tall as you are. Now, bend your arms and place your hands on your hips if it feels ok for you. Stand like this for a while [20 s. PAUSE]. Stand up straight almost like somebody is gently pulling your head up, so you are standing upright with a straight back. Make sure to keep your shoulders pulled back. Lift your chin and push your chest slightly forward [20 s. PAUSE]. While standing in this position, notice your face. Relax the muscles around your eyes and forehead. Now gently nod your head. Up and down. Up and down [20 s. PAUSE]. The exercise is now over.*

***Contractive [CON] body manipulation:***

*Start by standing up. Keep your shoulders relaxed and let your arms hang down the side of your body. Let your body collapse a little [20 s. PAUSE]. Now place your right hand on your left shoulder and your left hand on your right shoulder if it feels ok for you. Tuck your chin slightly. Stand like this for a while [20 s. PAUSE]. Make sure you are standing in a slouched position almost like somebody is gently pulling your head down. Make sure to keep your shoulders pushed slightly forward [20 s. PAUSE]. While standing in this position, notice your face. Relax the muscles around your eyes and forehead. Now gently shake your head. From side to side. From side to side [20 s. PAUSE]. The exercise is now over.*

***Active control [ACO] body manipulation:***

*Start by standing up. Stand in a position that feels comfortable and natural for you [10 s. PAUSE]. Now start walking in place [25 s. PAUSE]. Change your tempo so you are almost running in place [25 s. PAUSE]. Now switch to doing high knee lifts in place [25 s. PAUSE]. The exercise is now over.*

***Neutral [NEU] body manipulation:***

*Start by standing up. Stand in a position that feels comfortable and natural for you [20 s. PAUSE]. Make sure that your shoulders and arms fall naturally along the side of your body [20 s. PAUSE]. While standing in this position, notice your face. Relax the muscles around your eyes (…) and forehead (…), but don’t try to control them [20 s. PAUSE]. Keep standing in a position that feels natural to you [20 s. PAUSE]. The exercise is now over.*

**Depicted Action Tendencies (DAT) drawings.**

| Approach reward 1 | Approach reward 2 | Approach threat 1 | Approach threat 2 | Avoid Reward 1 | Avoid Reward 2 | Avoid threat 1 | Avoid threat 2 |
| --- | --- | --- | --- | --- | --- | --- | --- |
| 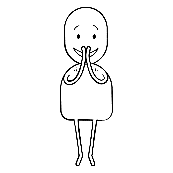 | 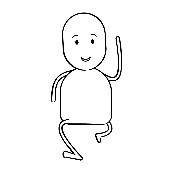 | 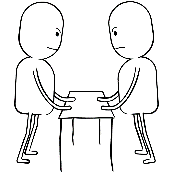 | 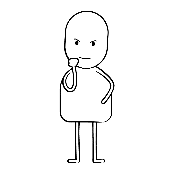 | 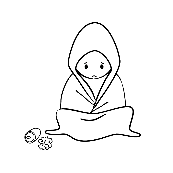 | 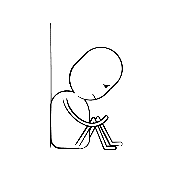 | 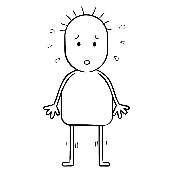 | 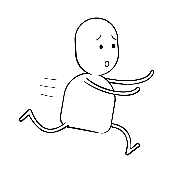 |

From: O'Toole, M. S., & Mikkelsen, M. B. (2021). Developing a non-verbal, self-report assessment tool of action tendencies: The Depicted Action Tendencies (DAT) instrument. *Scand J Psychol*, *62*(3), 289-300. <https://doi.org/10.1111/sjop.12710>

**Statistical Analyses**

Following the pre-registered analytic plan, multilevel analyses (MLMs) were conducted to test the three hypotheses. For all MLMs, time points (level 1) were nested within individuals (level 2). Four types of MLMs were conducted. Models 1-3 address Hypothesis 1 that participants who adopt an expansive posture would be more likely to take action and report more favorable affective outcomes than participants who adopt a contractive posture and participants in control conditions, and Hypothesis 2 that participants who adopt a contractive posture would be less likely to take action and report less favorable affective outcomes than participants who adopt an expansive posture and participants in control conditions. Model 4 address Hypothesis 3 that the effect of expansive and contractive postures on taking action will happen as a result of changes in affect. The four types of models are specified here:

1) To test whether body manipulations had an effect on taking action and affect assessed at T_3_, separate multilevel models for each of the outcomes (i.e., taking action, emotions, and action tendencies) at T_3_ were conducted with body manipulations (i.e., expansive, contractive, walking in place, neutral) as the predictor.

2) To test whether body manipulations had an effect on changes in affect from planning action to *right before* taking action, separate MLMs for each of the outcomes (i.e., emotions, action tendencies, and appraisals) were conducted with body manipulations, time (i.e., T_1_ and T_2_), and the interaction term (i.e., body manipulations × time) as predictors.

3) To test whether body manipulations had an effect on changes in emotions and action tendencies from planning action to *after* taking or not taking action, separate MLMs for each of the outcomes (i.e., emotions and action tendencies) were conducted with body manipulations, time (i.e., T_1_ and T_3_), and the interaction term (i.e., body manipulations × time) as predictors.

4) To test whether *changes* in affective outcomes from planning action (T_1_) to after the body manipulation instructions (T_2_) had an effect on taking action (T_3_), MLMs were first conducted to assess changes in each of the affective outcomes from T_1_ to T_2_, disregarding body manipulations. Significant results were followed by MLMs with the change score for the affective outcome (i.e., T_1_ to T_2_) as a predictor of taking action.

To explore whether the effects of body manipulations on taking action were moderated by motivational traits and psychopathology, explorative MLMs were conducted. Specifically, separate MLMs were conducted with body manipulations and each of the baseline measures (i.e., subscales of DASS, EROS, and BIS/BAS), and the interaction term (i.e., body manipulations × baseline measure) as predictors of taking action at T_3_. The *p*-values for the fixed effects in the MLMs were obtained from t-tests using a normal approximation of degrees of freedom. To reduce the risk of a Type I error, family-wise corrections of *p*-levels were employed for moderator analyses. Accordingly, a *p*-level of .017 (.05/3) was applied reflecting that group differences were assessed across three questionnaires (i.e., DASS, BIS/BAS, and EROS).

**Post-hoc Sensitivity Analysis**

A post-hoc sensitivity analysis was conducted to determine the smallest between-groups effects we could reliably detect with a sample size of 127 and a statistical power of 0.80. Using a conservative approach, we based the sensitivity analysis on a sample size of 127, and 8 repeated measures, indicating the average number of weeks (7.58) the participants completed a week and reported listening to the audio file with the body display instructions. For the weeks the participants listened to the audio file, the average correlation for the outcome *action as planned* was *r* = .156. Based on this information, the study was powered to detect effect sizes of Cohen’s *d* = 0.30 at *p* = .05, and *α* = 0.80.

**Robustness analyses**

Robustness analyses were conducted to assess the robustness of the results. Specifically, the analysis exploring the effect of body display instructions on taking action were reconducted after 1) omitting participants who did not complete all 12 weeks, 2) omitting participants who indicated they had knowledge about power posing, and 3) omitting participants who indicated they did not have knowledge about power posing.

After removal of participants who did not complete all 12 weeks (*N* = 15), the main analysis assessing the effect of body display instructions on taking action at T_3_ was reconducted with participants who completed all 12 weeks (*N* = 112). The results remained robust with no significant effect of body display instructions on taking action, *F* = 0.77, *p* = .511, *d* = 0.06.

After removal of participants who indicated they had knowledge about power posing (*N* = 52), the main analysis assessing the effect of body display instructions on taking action at T_3_ was reconducted with participants who indicated they had no knowledge of power posing (*N* = 56)^[[1]](#footnote-1)^. The results remained robust with no significant effect of body display instructions on taking action, *F* = 0.88, *p* = .445, *d* = 0.10.

After removal of participants who indicated they did not have knowledge about power posing (*N* = 56), the main analysis assessing the effect of body display instructions on taking action at T_3_ was reconducted with participants who indicated they had knowledge of power posing (*N* = 52). The results remained robust with no significant effect of body display instructions on taking action, *F* = 0.99, *p* = .397, *d* = 0.10.

1. Note: Participants reported on knowledge about power posing upon completing *all* 12 weeks of the study. As such, data were missing for participants who did not complete all 12 weeks of the study (*N* = 15). Furthermore, due to a technical error, four additional participants were not asked to report on knowledge of power posing. [↑](#footnote-ref-1)
